# Pitfalls in Photochemical and Photoelectrochemical Reduction of CO_2_ to Energy Products

**DOI:** 10.3390/molecules29194758

**Published:** 2024-10-08

**Authors:** Tomasz Baran, Domenico Caringella, Angela Dibenedetto, Michele Aresta

**Affiliations:** 1 Innovative Catalysis for Carbon Recycling-IC^2^R, Via Camillo Rosalba 49, 70124 Bari, Italy; tomasz.baran@gmail.com (T.B.); circsrl13@gmail.com (D.C.); 2 Interuniversity Consortium on Chemical Reactivity and Catalysis (CIRCC), Via Celso Ulpiani 27, 70126 Bari, Italy; 3 Department of Chemistry, University of Bari Aldo Moro, 70125 Bari, Italy

**Keywords:** photochemical reduction of CO_2_, photoelectrochemical reduction of CO_2_, CO2RPs, false positive, risk management

## Abstract

The photochemical and photoelectrochemical reduction of CO_2_ is a promising approach for converting carbon dioxide into valuable chemicals (materials) and fuels. A key issue is ensuring the accuracy of experimental results in CO_2_ reduction reactions (CO2RRs) because of potential sources of false positives. This paper reports the results of investigations on various factors that may contribute to erroneous attribution of reduced-carbon species, including degradation of carbon species contained in photocatalysts, residual contaminants from synthetic procedures, laboratory glassware, environmental exposure, and the operator. The importance of rigorous experimental protocols, including the use of labeled ^13^CO_2_ and blank tests, to identify true CO_2_ reduction products (CO2RPs) accurately is highlighted. Our experimental data (eventually complemented with or compared to literature data) underline the possible sources of errors and, whenever possible, quantify the false positives with respect to the effective conversion of CO_2_ in clean conditions. This paper clarifies that the incidence of false positives is higher in the preliminary phase of photo-material development when CO2RPs are in the range of a few 10s of μg g_cat_^−1^ h^−1^, reducing its importance when significant conversions of CO_2_ are performed reaching 10s of mol g_cat_^−1^ h^−1^. This paper suggests procedures for improving the reliability and reproducibility of CO2RR experiments, thus validating such technologies.

## 1. Introduction

Research on the photochemical (PC) and photo(electro)chemical (PEC) reduction of CO_2_ is gaining momentum worldwide because it proposes an innovative solution to combat climate change for sustainable development [1]. Photocatalytic reduction enables the conversion of carbon dioxide and water, two greenhouse gases (GHGs), into added-value chemicals and fuels directly using sunlight as the primary energy source, a process that may contribute to shifting away from fossil carbon, a necessity unanimously agreed on at COP28 [2]. Therefore, photocatalytic and photo(electro)catalytic reduction of CO_2_ are major research topics in the field of carbon dioxide capture and utilization (CCU) [3]. Key research topics are in the direction of engineering new stable photocatalysts, enhancing light efficiency, and developing scalable technologies that can be applied at the industrial level. Transforming CO_2_ into valuable products (chemicals, materials, fuels) also contributes to the implementation of a carbon circular economy [4], leaving the linear economy model based on fossil C used for over 200 years.

**Figure 1:**
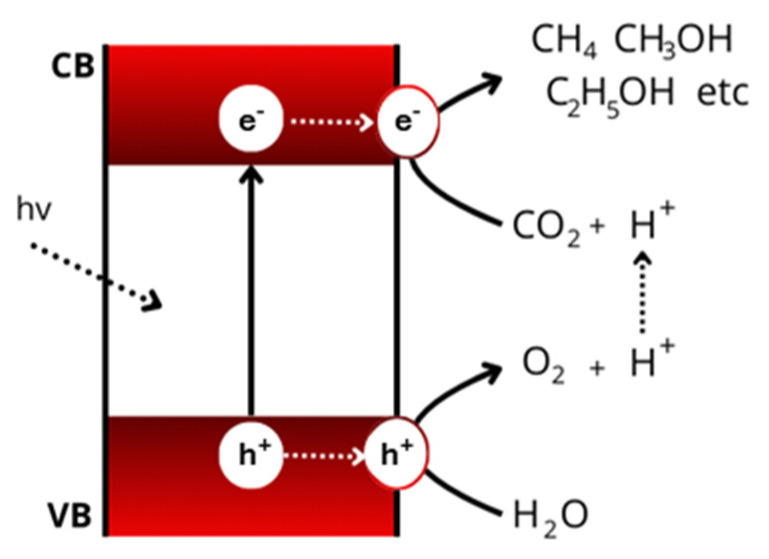
A semiconductor at work. Abbreviations: CB—conduction band; VB—valence band; hv—photon. The drawing represents a simplified process scheme.

Key actors in PC and PEC processes are semiconductors. Light absorption by a semiconductor leads to charge separation with the formation of “electron–hole pairs”. The excited electrons and holes then participate in redox reactions in which CO_2_ and water are implied, leading to the reduction of CO_2_ and the formation of C_1+_ compounds (such as carbon monoxide, formaldehyde, methane, methanol, ethanol, and others) from one side and water oxidation from the other with the production of oxygen [5] (Figure 1).

**Figure 2:**
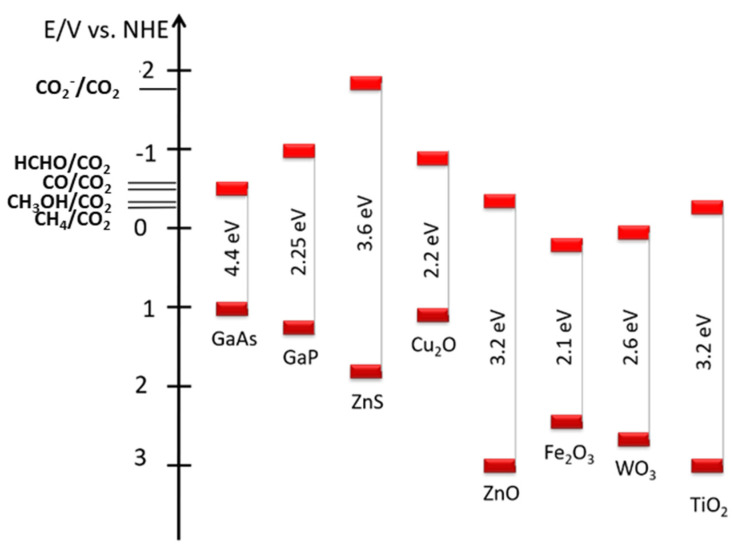
Semiconductors band gap compared to the reduction potential of CO_2_ to a variety of products in water.

Semiconductors are classified according to their band gap (BG) that should ideally match the potential of reduction of CO_2_ (CO2RR) into hydrogenated products (Figure 2).

In CO2RRs, a variety of materials are used, among which copper oxides (*p*-type), or titanium or zinc oxides (*n*-type), are often enhanced with co-catalysts or dopants to improve their efficiency [6] through charge separation stabilization or fastening of charge surfacing. Moreover, in order to increase the lifetime or optimize the adhesion of photomaterials to electrodes, from more traditional ones (TiO_2_) to novel metal–organic frameworks (MOFs) and perovskites, a number of organic materials are used [7,8,9,10,11,12,13,14,15]. Catalyst design plays a key role in reaching an efficient CO2RR and a deeper understanding of reaction pathways and factors influencing photocatalytic activity.

However, photomaterial manipulation often contains hidden traps linked to the response of hybrid species to radiations, and the results of their use are often questioned [16,17,18] because residual organics or added components used to enhance the photomaterial activity or adhesivity to electrodes can undergo degradation and be the source of species erroneously counted as CO2RPs [17,19,20,21,22].

In this paper, we categorize the classes of errors, showcased with a number of commonly used materials and experiments, and highlight the importance of careful control experiments, standardization protocols, blank tests, and the use of isotopically labeled CO_2_ to confirm the definitive origin of the reaction products [16,19,23] for advancing the field of CO2RR. Obtaining genuine CO2RPs and avoiding products derived from serendipitous carbon species requires rigorous experimental conditions and analytical methods to validate findings.

Therefore, this article comprehensively analyzes traps and pitfalls we have encountered during our research on photocatalytic CO_2_ reduction. Some of them are common to other studies on CO_2_ conversion, as reported in the literature, and others are new. By highlighting such challenges and proposing control methodologies, we aim to stimulate other researchers’ sensitivity towards more reliable and reproducible results, making the photo(electro)catalytic systems in CO2RRs more robust.

## 2. Analysis of Potential Sources of False-Positive Results and Quantification of Their Relevance

In this section, we itemize and discuss potential exogenous sources of CO2RR that may produce reduced-carbon species (C_1_ and C_n_ products) and generate false positives. The relevance of such risk is higher when photo(electro)catalysts are in their early test phases and, most frequently, produce amounts of products comparable to that of species originated from contaminants or the decomposition of materials used ad hoc (in the order of 5-50 μg g_cat_^−1^ h^−1^). As a general consideration, it must be said that PC and PEC experiments differ in the fact that the latter allows for separating the reduction and oxidation processes, while the former usually does not. Therefore, oxygen formed upon water oxidation may retro-react with organics with an overall lower yield of products. Oxygen must be avoided in the cathodic compartment in PEC experiments as it may produce reactive oxygen species under irradiation, such as singlet oxygen, superoxide anion radicals, hydroperoxide radicals, hydroxyl radicals, and others [24], which can attack materials used in devices, leading to the formation of simple organic molecules, potential CO2RPs.

The sources of false positives discussed in this paper include (i) the degradation of carbon-containing photocatalysts, (ii) the presence of contaminants on the photocatalyst stemming from their synthesis, such as residual surfactants and chemicals as reagents or solvents, (iii) the decomposition of sacrificial reagents or reaction additives, (iv) contaminants from other system components, including the photoreactor or laboratory glassware, (v) airborne contaminants within the laboratory environment, and (vi) the operator. These factors must be meticulously controlled and accounted for to ensure the accuracy and reliability of the experimental outcomes in carbon dioxide reduction experiments.

### 2.1. Degradation of Carbon-Containing Photocatalysts

Intensive research in the field of photocatalytic reduction has led to the investigation of an extraordinarily broad range of photocatalytic materials, including traditional inorganic materials, primarily metal oxides [6,25,26], sulfides [5,27], iodides [28], selenides, and tellurides [29]. Additionally, carbon-containing materials are of significant interest, such as metal–organic frameworks (MOFs) and covalent organic frameworks (COFs) [14]; graphene and graphene oxide-based materials [30]; carbon dots and nanotubes [31]; graphitic carbon nitride and related compounds [15]; and homogeneous photocatalysts based on metal complexes with organic ligands [32,33]. Furthermore, surface-modified semiconducting materials incorporating or even covered with complex compounds, such as dyes and other sensitizers, are being extensively explored [34,35,36,37]. Such a broad array of materials reflects the effort to enhance the efficiency and effectiveness of photocatalytic reduction processes. On the other hand, the degradation of carbon-containing materials by photogenerated holes or electrons can lead to the formation of simple C_1_ compounds (such as CO, CH_2_O, CH_3_OH, or CH_4_), or even C_n_ species, which can be erroneously identified as products of CO_2_ reduction. Such degradation processes can significantly impact the accuracy of experimental results with an overestimation of the efficiency of the CO2RR.

**Figure 3:**
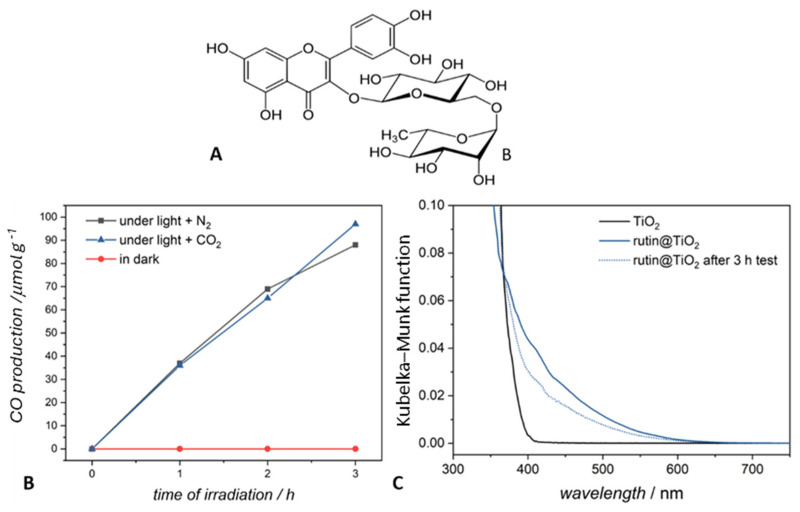
(**A**) Structure of rutin. (**B**) Evolution of carbon monoxide during the irradiation of rutin@TiO_2_ in suspension under N_2_ (black curve) and CO_2_ (blue curve). (**C**) Kubelka–Munk function from diffuse reflectance spectra of TiO_2_—P25 Degussa and TiO_2_ modified with rutin. The dotted line shows the spectrum of the material after 3 h of irradiation.

As a first example, we discuss the case of TiO_2_ functionalized with rutin (rutin@TiO_2_), an organic sensitizer used to shift the light absorption range towards visible light [25,38,39,40,41]. The structure of the sensitizer and the spectroscopic features of rutin@TiO_2_ are given in Figure 3A. Irradiation of a suspension of rutin@TiO_2_ in double-distilled and deionized water under a nitrogen atmosphere leads to the formation of carbon monoxide, as demonstrated by the gas chromatographic analysis of the gas phase (Figure 3B, black curve). The observed evolution of CO is almost linear during the first two hours of the experiment and continues for hours, reaching values of over 80 μmol/g_cat_^−1^ over three hours when it starts to decrease. Because of the lack of other sources of carbon, it is evident that CO derives from the degradation of rutin. Noteworthily, such production of CO is comparable to the yield in the presence of CO_2_ (97 μmol/g_cat_^−1^) (Figure 3B, blue curve) over the same time interval. Interestingly, after two hours of irradiation, the CO production from rutin started to flatten, indicating that most of the rutin present in the photocatalyst sample was degraded. In Figure 3C, the spectrum of irradiated materials (3 h) is compared with that of pristine materials and TiO_2_. Spectroscopic analysis shows a decrease in the intensity of the band related to charge transfer between TiO_2_ and rutin [40], proving the degradation of the latter. It is worth mentioning that the literature reports that rutin@TiO_2_ can form reactive oxygen species (ROS) under light [39]. As mentioned above, such ROS species are strong oxidants and can react with the rutin molecule, leading to its degradation. The irradiation of rutin@TiO_2_ in the absence and presence of CO_2_ thus causes the degradation of the organic molecule, which is clearly shown by the modification of its UV-Vis spectrum after 3 h of irradiation and by the production of CO detected in the gas phase in equilibrium with the condensed phase in the absence of CO_2_.

Metal acetylacetonates (e.g., titanium(IV)acetylacetonate, iron(III)acetylacetonate, gold(III)acetylacetonate, or zirconium(IV)acetylacetonate) are often used as photocatalysts or precursors of photocatalysts [42,43,44]. Noteworthily, the thermal decomposition of acetylacetonates is often used to form oxides or other desirable materials, with precise control of both the deposition and growth processes.

Some acetylacetonates are also used for the deposition of oxide thin films using chemical vapor deposition (CVD) or atomic layer epitaxy (ALE) methods. Obviously, the organic part is destroyed in such processes. It is known that the keto–enol structures of acetylacetone affect its photophysical and photochemical properties [45]. However, it has been found [45] that the irradiation of acetylacetone or its metal derivatives leads to the degradation of pristine materials, with the formation of formic acid, acetic acid, carbon monoxide, and formaldehyde (Table 1) that can mistakenly be identified as CO_2_ reduction products when photomaterials prepared from acetylacetone are used as photocatalysts.

Additionally, to enhance the efficiency of photoelectrochemical (PEC) reactions, various polymers with extended π-conjugated electron systems are utilized as photocatode and photoanode modifiers that may allow for the high mobility of charge carriers [46,47], enhancing conductivity while improving stability and the overall photoelectrochemical response. Polymers like Nafion^®^ are used as binders or additives to improve the mechanical stability and ionic conductivity between photomaterials and the conductive support, thus ensuring an efficient charge transfer, which is essential for efficient photoelectrochemical reactions.

Commonly used polymers are polyaniline (PANI) and poly(3,4-ethenedioxythiophene) (PEDOT), which are valued for their high electrical conductivity and environmental stability in a broad range of conditions when used with photomaterials like CdS, TiO_2_, Cu_2_O, and ZnO [47,48,49,50]. Polyetheneimine (PEI) is used for its strong adhesive properties and ability to enhance the adsorption of light, reduce the recombination rate of photogenerated carriers, and improve the overall charge transfer process, thereby boosting photoelectrochemical efficiency [51,52,53].

However, it must be highlighted that all the above-mentioned polymers can undergo hydrolytic, thermal, chemical, and photochemical degradation under use.

**Figure 4:**
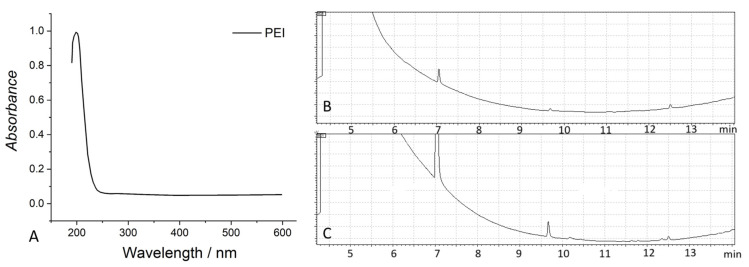
(**A**) UV-Vis spectrum of PEI dissolved in hot water. The spectrum was obtained by using the double-beam spectrophotometer Shimadzu UV2700i (Shimadzu, Kyoto, Japan). (**B**) Products revealed by GC upon irradiation of PEI in the gas phase and in water solution (**C**) under a N_2_ atmosphere.

In particular, they can be degraded by reactions with photogenerated reactive oxygen species [54]. PEI absorbs only UV light (see the spectrum in Figure 4A), and thus cannot be easily degraded by irradiation with visible light. Nonetheless, commonly used light sources, such as XBO or HBO lamps or even the solar spectrum itself, include a small portion of UV radiations. They can cause the degradation of PEI, both in the gas phase and when suspended in water, either by direct oxidation/reduction by photogenerated charges or reaction with generated reactive oxygen species. C_1_ and C_n_ carboxylic acids, aldehydes, and ketones, which can erroneously be considered CO2RPs, are formed (Figure 4B,C). In addition, amines and imines are produced in the presence of water because of photo-induced hydrolysis.

Therefore, such chemicals that should stabilize/activate the photocatalysts are not innocent, and strict operative conditions must be set in order to avoid their decomposition under irradiation-producing species that can be counted as CO2RPs.

### 2.2. Contaminants on a Photocatalyst

Contaminants on a photocatalyst (surface and bulk) may lead to misleading conclusions, compromising the reliability of experimental results and hindering the advancement of research in photocatalysis. It often happens that during the synthetic process, organic compounds such as surfactants, ionic liquids, organic precursors, or organic solvents are used for a variety of operations. If the produced photocatalytic materials are not properly rinsed and organics are not completely eliminated, the latter may constitute a source of species mistakenly labeled as CO_2_ reduction products. We have verified (vide infra) that residual fatty acids, surfactants, and ionic liquids irradiated in the presence of a photocatalyst, under the same reaction conditions in which CO_2_ is reacted, can produce methanol, ethanol, C_3_ species, and other more complex molecules. Even, serendipitous organics can decompose under photochemical or photo(electro) chemical conditions or act as sacrificial hole scavengers, producing false positives in CO2RRs.

Vigilance during synthesis and meticulous purification protocols are essential to minimize the impact of impurities on CO2RRs. Thorough characterization and analysis techniques are indispensable for identifying and quantifying any contaminants present on the photocatalyst surface. Understanding the potential sources of interference enables the development of strategies for mitigating their effects and improving the reliability and reproducibility of experimental findings in photocatalytic studies. We discuss some specific cases in the following paragraphs.

#### 2.2.1. Surfactants

Surfactants are commonly used in the synthesis of photocatalysts [55] for the following reasons: (i) control of particle size and morphology; (ii) prevention of aggregation; (iii) stabilization of colloidal suspensions; (iv) surface modification; and (v) control of crystal phase and orientation. Surfactants are generally washed off the surface of powdered photomaterials using water or suitable solvents. However, the completeness of removal depends on a variety of factors such as the type of surfactant used, the nature of the material surface, and the specific synthetic conditions. Unwashed residues of surfactants can serve as a source of carbon in competition with CO_2_ in a CO2RR and may lead to false results.

**Figure 5:**
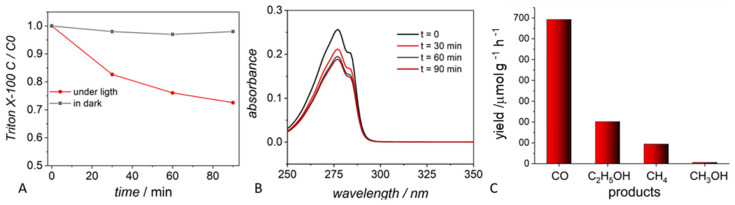
Photodegradation of residual Triton-100 using a Xe lamp. (**A**) Trends in the photocatalytic degradation of Triton-100 with time in the absence of CO_2_. (**B**) Photodegradation of Triton-100 followed by UV-Vis spectroscopy. (**C**) Products observed in a blank test, by irradiating a sample of ZnS containing residual Triton-100 under nitrogen. The Y-axis reports the concentration of species.

Triton is a known organic nonionic surfactant used in the synthesis of nanomaterials such as CuO, SiO_2_, Ag, ZnS, CdS, and Fe_2_O_3_ [56,57,58,59,60]. ZnS nano-powder prepared in the presence of Triton X-100, according to the literature methods, was tested in our laboratory in a blank test in a CO2RR. A suspension of ZnS under a dinitrogen atmosphere (with the exclusion of CO_2_) was irradiated with a Xe-lamp for the time usually used in a CO2RR and was shown to produce carbon monoxide, methane, methanol, and ethanol (Figure 5). Such compounds were not formed during a test performed in the dark, confirming the role of light in Triton decomposition. Therefore, C_1_ and C_2_ products were formed under the photocatalytic degradation of residual organics present on the photocatalyst. The degradation of residual Triton X-100 was also easily monitored using UV-Vis spectroscopy, as shown in Figure 5B, following the lowering of the band at 277 nm during irradiation. Such a band can be assigned to the π→π* transition characteristic of aromatic systems. During the 90-minute test, the absorbance of the band at 277 nm was significantly reduced. The mechanism of Triton photocatalytic decomposition can be found in the literature [61]. The concentration of Triton was evaluated using the Lambert–Beer law. Figure 5A shows that during 90 min of irradiation, ca. 30% of the initial Triton was degraded. In the dark, the material was stable with only a very marginal change in concentration.

#### 2.2.2. Ionic Liquids

The unique properties of ionic liquids (ILs) make them valuable actors in the synthesis of photocatalytic nanomaterials, enabling precise control over structure, properties, and performance in various applications [62]. Ionic liquids can act as solvents or reaction media in the synthetic process; indeed, they offer several advantages over traditional solvents, such as low volatility, higher thermal stability, and tunable properties [62,63]. These characteristics make ILs suitable for controlling reaction conditions and facilitating the formation of well-defined nanomaterials. Moreover, ILs can also serve as templates or structure-directing agents in the synthesis of photocatalytic nanomaterials [64]. By carefully selecting the composition and properties of ILs, it is possible to influence the morphology, size, and crystallinity of the resulting nanomaterials. Ionic liquids can also stabilize nanoparticles and prevent their agglomeration or undesired growth during synthesis. Finally, ILs can modify the surface properties of nanomaterials, such as enhancing dispersibility, controlling surface charge, or introducing functional groups [65]. These modifications can improve the stability, reactivity, and performance of photocatalytic nanomaterials.

**Figure 6:**
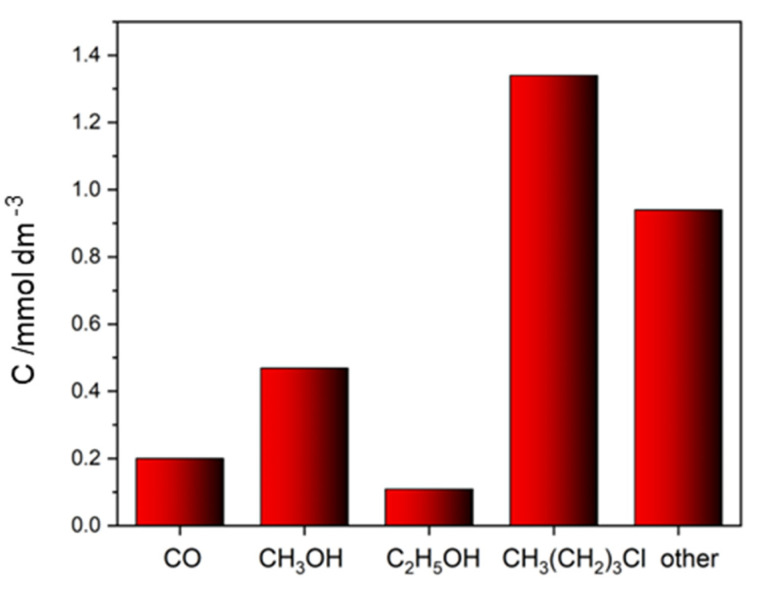
Products formed during the photoirradiation (Xe-lamp) of residual 1-butyl-3-methyl imidazolium chloride for 1 h in water in the presence of CuO/ZnO. The Y-axis reports the amount of formed products.

The miscibility of ILs with water depends on their structure. Some ionic liquids may be well soluble in water, while others may be only sparingly soluble, insoluble, or form a microemulsion [66]. However, they can be hard to wash off the surface of the photocatalyst. Unwashed residuals of ILs can undergo photocatalytic decomposition, leading to false positive results. We performed a photocatalytic blank test of the degradation of the residual ionic liquid 1-butyl-3-methylimidazolium chloride (a known compound used for the synthesis of nanomaterials [67]) in the presence of a CuO/ZnO photocatalyst. The GC analysis of the gas and condensed phases showed the formation of methanol, ethanol, carbon monoxide, hydrogen, and a few other compounds, such as chlorobutane, as products of IL degradation (Figure 6). Such results clearly demonstrate that unwashed traces of 1-butyl-3-methylimidazolium chloride undergo a photocatalytic degradation with the formation of numerous products that might be counted as produced in a CO2RR.

#### 2.2.3. Solvents

**Figure 7:**
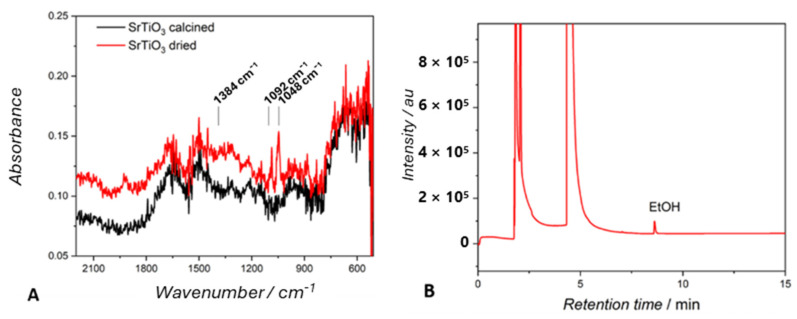
(**A**) FTIR spectra of dried (red) and calcined (black) SrTiO_3_. (**B**) GC-BID analysis of the headspace from the photocatalytic test performed in a suspension of dried SrTiO_3_ in an inert atmosphere with an irradiation time of 1 h and white light.

Organic solvents are often used in synthetic processes to both prepare photocatalytic nanofilm nanoparticles and effectively remove residues of reagents. For example, one can consider the synthesis of titanium-based materials, in which titanium(IV)isopropoxide, tetrabutyltitanate, or titanium(IV)butoxide are used as precursors [26,68]. In such cases, organic solvents like ethanol are frequently employed to wash the prepared materials. We prepared SrTiO_3_, a known photocatalyst for CO_2_ reduction [26,69], from tetrabutyltitanate in water–ethene glycol (See the Materials Section). The target photocatalyst was isolated, washed with ethanol, and dried under vacuum to eliminate residual Ti compounds and ethene glycol. The dried material was analyzed using FTIR spectroscopy. The spectrum (Figure 7) showed not only peaks corresponding to strontium titanate but also, despite drying, a few typical bands of ethanol, at 1384, 1092, and 1048 cm^−1^. Noteworthily, when photocatalytic tests were performed using a suspension of dried SrTiO_3_ in a N_2_ atmosphere, the formation of ethanol was observed at a rate of 0.2 mmol g^−1^ h^−1^, a value that is comparable to usual yields for CO2RRs.

To demonstrate the origin of such ethanol clearly, the dried-SrTiO_3_ was calcined at 300 °C, and the FTIR of the calcined material did not show any band attributable to alcoholic functionalities (Figure 7A, black trace), and a blank test under N_2_ did not show ethanol.

In several reports, a solution of Nafion in isopropanol is used to obtain higher-quality photoelectrodes during the deposition of powders onto FTO glass or other substrates. In our research, we used graphitic carbon nitride (*g*-C_3_N_4_) as a photocatalyst and light harvester, and for thin-film preparation, we used Nafion in isopropanol. Following the literature procedure, the glass plates covered with the material were first dried at room temperature, then in an oven at 80 °C (boiling temperature of isopropanol), and, finally, at 150 °C for 2 h. The so-produced thin films were used in photocatalytic blank tests. As summarized in Table 2, GC analyses showed the presence of isopropanol in each test. Even heating at 150 °C did not completely eliminate isopropanol from *g*-C_3_N_4_.

Therefore, blank tests should be used to demonstrate that photomaterials are free of residual organics that would erroneously be counted as CO2RPs.

### 2.3. Decomposition of Sacrificial Reagents or Reaction Additives

Water is the only economically viable reducing agent in the photocatalytic reduction of CO_2_, providing both electrons and protons necessary for the synthesis of hydrogenated species such as hydrocarbons and their derivatives. However, because of kinetic and thermodynamic constraints, the use of pure water remains a significant challenge. Many photocatalysts are unable to oxidize water because their valence band edge potential is not sufficient. Consequently, inorganic and organic sacrificial hole scavengers (triethylamine, triethanolamine, ascorbic acid, propanol) are often used in research [70,71]. It is important to note that while such scavengers supply electrons and hydrogen atoms for CO_2_RRs, under irradiation, they can originate organic compounds, mistakenly identified as CO2RPs.

Glycerol can be used as a sacrificial hole scavenger in photocatalytic tests [40,72]. Glycerol, like other sacrificial electron donors, provides protons while undergoing oxidation itself. Potential degradation products of glycerol include hydrogen, formic acid, formaldehyde, acetic acid, carbon dioxide, methanol, and 1,3-dihydroxyacetone. We performed an experiment in which a suspension of ZnS, a well-known photocatalyst for CO_2_ reduction [5,73], in a water–glycerol solution was irradiated in a N_2_ atmosphere in an NMR tube. The first product of glycerol oxidation was 1,3-dihydroxyacetone, which was determined using NMR (^1^H resonances found at 4.35 ppm, as compared with the literature, 4.40 ppm [74]). Furthermore, formic acid was identified, evidenced by a ^1^H-NMR signal at 8.25 ppm [75]. The latter compound, more easily than the former, could be mistakenly identified as a product of carbon dioxide reduction on the surface of zinc sulfide [5]. These data highlight the necessity of careful analysis to distinguish between true products of photocatalytic CO_2_ reduction and those arising from the oxidation of sacrificial electron donors to ensure the accuracy and reliability of the experimental results.

### 2.4. Contaminants from Other System Components, Including the Photoreactor or Laboratory Glassware, Equipment, and Materials Used, Such as Pipettes, Syringes, Vials, and Filtration Materials

Equipment and devices can generate pollutants per se. An example of such devices is 3D printers, which are increasingly finding applications in laboratory work. It has been demonstrated that printing using filaments such as ABS, HIPS, or PET leads to the emission of a range of compounds including styrene, hexanal, acetophenone, ethylbenzene, benzene, methanol, pinene, octanal, toluene, nonanal, pentanol, butanol, propylene glycol, acetic acid, and others, depending on the filament used [76,77].

Laboratory plasticware such as test tubes or pipette tips are used routinely in most laboratories. It has been demonstrated that various agents used in manufacturing can leach from these plasticwares into an aqueous or organic solution, thus affecting the investigated reaction [78,79].

Analytical methods can be also affected by numerous factors related to the used laboratory materials and equipment. Gaseous samples can be contaminated by compounds released from syringes or sampling bags (e.g., Tedlar bags) used for GC analysis. Mochalski et al. [80] reported that Tedlar bags emitted several compounds including dimethylacetamide, phenol, carbon disulfide, carbonyl sulfide, n-hexane (and other hydrocarbons), 2,4-dimethyl heptane, 4-methyl octane, acetonitrile, and 1-methoxy-2-propylacetate [81].

An effective preventive measure is to set the entire chain of equipment for the application of photochemical and photoelectrochemical catalysts, including the PEC cell, and for product analyses (FTIR, UV-Vis, GC, GC-MS) in an “*organic-free laboratory*” dedicated to CO2RRs, thus avoiding contamination by other operations in the laboratory. This experiment was successfully carried out within the EU-DESIRED Project at our facilities and we can assert that indeed the use of a dedicated laboratory and equipment to CO2RRs with the exclusion of any other synthetic operation including organics changes the quality of data and cuts out potential false positives, making found results reliable and repeatable.

### 2.5. Airborne Contaminants within the Laboratory Environment

The air in a laboratory where chemical compounds and solvents are used can be highly contaminated, as described in detail elsewhere [82,83]. Such contaminated air can be absorbed by solvents and reaction mixtures and become the source of a variety of C-based compounds that can erroneously be counted as CO2RPs.

### 2.6. The Operator

The operator can be a potential source of sample contamination, leading to false positive results. In exhaled human breath, various volatile organic compounds (VOCs) can be found, though in trace amounts. These compounds include substances like oxygen-containing compounds (acetone, methanol, ethanol, etc.), hydrocarbons (methane, ethane, pentane, isoprene), sulfur-containing compounds (ethyl mercaptane, dimethyl disulfide, dimethyl sulfide), and nitrogen-containing compounds (ammonia, dimethylamine, trimethylamine) [84]. The concentrations of VOCs in exhaled breath are typically very low, often measured in parts per billion (ppb) or even parts per trillion (ppt). However, their concentration can unexpectedly increase depending on the operator’s health conditions and metabolic process alteration and affect samples under analyses if they are exposed during preparation/measurements [84].

**Figure 8:**
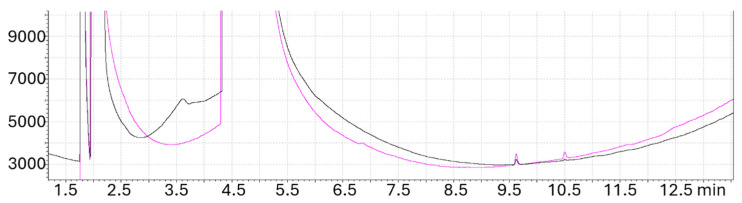
GC-BID analysis of blank tests carried out under a N_2_ atmosphere that show the presence of H_2_ (rt 3.65) and organics (rt 9.65 and 10.5) derived from the breath of two operators (black and pink curve) at the same exposure time (30 min) necessary for setting up the PEC equipment.

Using the standard chromatographic setup (see Materials and Methods Section) we use in work related to CO_2_ reduction, we carried out a careful test during one full month and with a set of four operators to verify to which extent the breath of laboratory operators can generate compounds that can mistakenly be counted as CO2RPs. The GC-BID analysis (Figure 8) revealed in samples under analysis the presence of ammonia, acetone, ethanol, and other organic compounds. Noteworthy, we verified that working under pure photochemical conditions in a controlled gas phase produces less organics than when using a three-phase PEC system where the solvent can act as a concentrator of external organics even because of a longer contact time with the operator during the assembly of the various parts of the PEC cell. Two of the most common false positives are constituted by ethanol and acetone, the latter emitted by operators having some metabolic diseases. The concentration of such exogenous chemicals can reach several ppm in a water electrolyte during the preparation of experiments if the latter is not very well confined and kept out of the contact with air. Therefore, the contact of equipment with the operator must be kept under strict control and blank tests must be used for verifying the origin of claimed CO2RPs.

## 3. Good Practices in CO_2_ Reduction

### 3.1. Detection and Elimination of Possible External Carbon Sources and Identification of Contaminants

Detecting and eliminating organic contaminants and any external carbon sources from the surface of photocatalysts, solvents, and equipment is crucial for removing uncertainties and ensuring reliable results. As we discussed above, such contaminants can originate from various sources; therefore, very accurate detection methods and removal techniques must be used.

Several analytical techniques can be used to detect organic contaminants. X-ray photoelectron spectroscopy (XPS), Fourier Transform InfraRed spectroscopy (FTIR), and energy-dispersive X-ray spectroscopy (EDX) can be used directly on a solid sample. Gas Chromatography–Mass Spectrometry (GC-MS) can be advantageously applied in desorption tests; thermal gravimetric analysis (TGA) can measure the weight loss of a photomaterial upon heating; and liquid- and solid-state NMR can be used to test solids and dissolved materials; among others.

**Figure 9:**
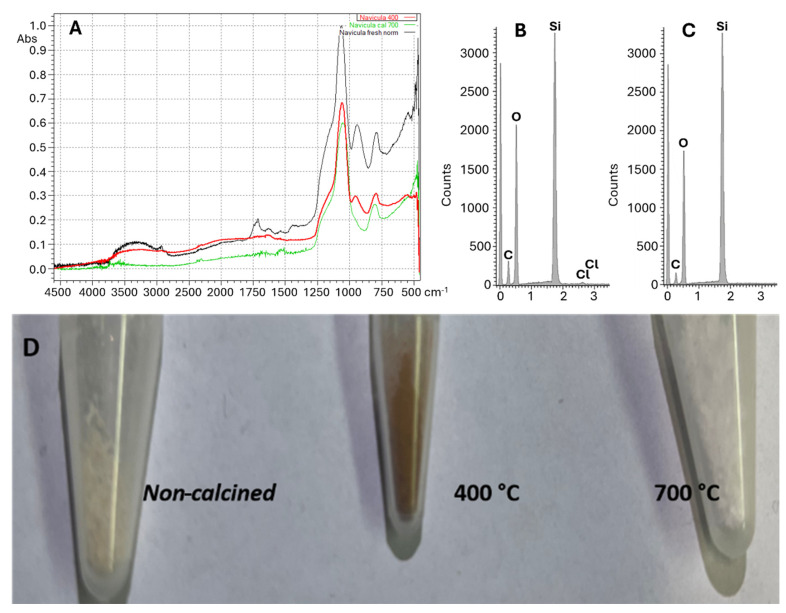
(**A**) FTIR of non-calcinated (black trace) frustules and frustules calcined at 400 °C (red trace) and 700 °C (green trace). (**B**) EDX of non-calcined frustules: the signal of carbon is well evident. (**C**) EDX of frustules calcined at 400 °C: the signal of carbon is reduced in intensity. (**D**) Photos of non-calcined frustules and frustules calcined at 400 °C and 700 °C.

Very recently, we investigated frustule-supported photocatalysts [85]. Our initial results indicated the reduction of carbon dioxide to methanol, acetone, and other compounds. However, by using FTIR (Figure 9A) and EDX analyses (Figure 9B,C), we found that frustules, being of biological origin (they are the siliceous skeleton of microalgae diatoms), contained residual organic carbon, regardless of the acid treatment they underwent during their preparation, and this raised questions about the origin of observed organics. To eliminate interference, we employed high-temperature annealing that causes pyrolysis and complete elimination of all pristine organic compounds. Mass loss measurements during calcination exceeded 30–50% of the initial mass. Figure 9D shows a photo of pristine frustules (whitish) and frustules calcined at 400 °C (brown powder) and 700 °C (white powder). Progressive elimination of organic carbon was also proved by EDX (compare Figure 9B,C). Calcination can be an easy but effective method to eliminate organic carbon contaminations from thermally stable samples. However, it should be highlighted that not all materials can be thermally treated; some require a protective atmosphere. J. You et al. proposed using an oxygen plasma treatment as an effective method for removing carbon contamination from solids and eliminating false positives in CO_2_ reduction [20]. Moreover, purified catalysts should be stored in a clean atmosphere, such as in a N_2_ atmosphere, to exclude the possibility of re-contamination. The use of a laboratory glove box to manipulate catalysts and carry out reactions is an advantageous solution.

Figure 9A clearly shows that heating frustules can cause the loss of organics and water produced in the conversion of terminal -Si-OH moieties (disappearance of the band at 950 cm^−1^) into -Si-O-Si- moieties (band at 850 cm^−1^).

In addition to thermal annealing and plasma treatment, to eliminate organic contaminants from the surface of photo-materials, the following methods can be used:

- Acid or base etching.
- Ultrasonic cleaning.
- Solvent (water) washing or sequential washing using a series of different solvents to target a broader range of contaminants. Residual organic solvents must then be carefully eliminated.
- UV–ozone cleaning.

Equal attention must be given to cleaning substrates used to deposit materials (in photo-electrochemistry), such as silicon wafers, FTO glass, ITO glass, metal foils, and carbon paper. The cleaning of such substrates can be performed by using chemical cleaning (RCA); a three-step procedure (SC-1, SC-2, and HF-dip) for removing organic and metallic contaminants and oxides [86]; or plasma cleaning, mechanical polishing, chemical cleaning by soaking in a mixture of HCl and water or in a piranha solution (a mixture of sulfuric acid and hydrogen peroxide). A blank test (under N_2_, both under irradiation and in the dark) is recommended to check for the presence of residual organics.

### 3.2. Experiments with Labeled ^13^CO_2_

Understanding the precise mechanisms and pathways involved in the CO_2_ reduction process is challenging. One powerful tool that has emerged to address these challenges is the use of isotopic ^13^CO_2_ for tracing CO2RPs. The natural abundance of ^13^C in C-based products is only 1.109%. It is, thus, easy to distinguish products derived from ^13^CO_2_ used as a reagent from products derived from any contaminant (biomass or fossil C-derived chemicals). The incorporation of ^13^C into CO2RPs can be traced using a variety of analytical techniques, among which FTIR, nuclear magnetic resonance (NMR), and mass spectrometry (MS) are the most at hand. The primary advantage of using ^13^CO_2_ is its ability to provide detailed mechanistic insights by tracking its incorporation into reaction products and intermediate species with high precision and even using operando techniques. Such understanding helps to highlight the pathway of CO_2_ reduction and excludes false positives, identifying products that do not originate from ^13^CO_2_ reduction. This leads to a more accurate assessment of catalytic efficiency, activity, and product validation.

**Figure 10:**
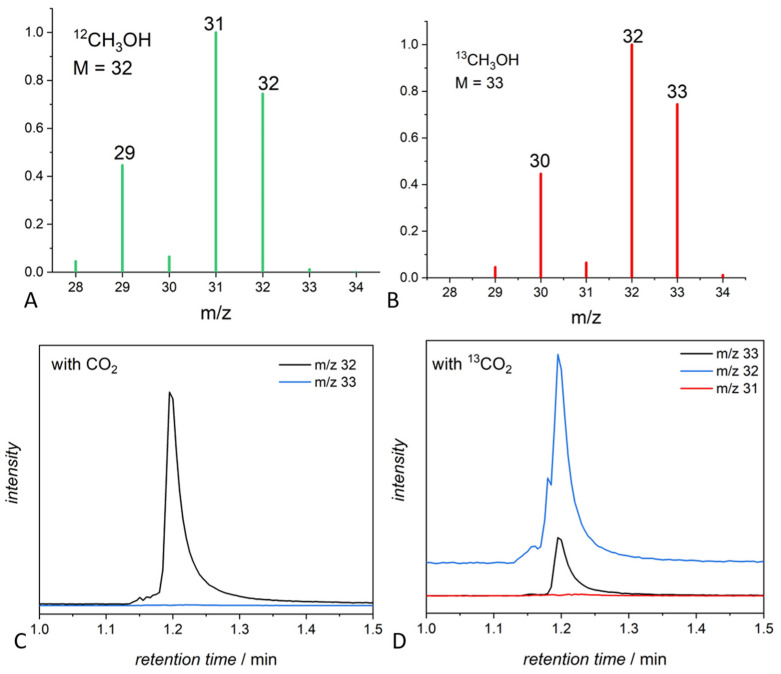
MS spectra of methanol ^12^CH_3_OH (**A**) and ^13^CH_3_OH (**B**). GC-MS-extracted ion chromatogram of ^12^CH_3_OH (**C**) and ^13^CH_3_OH (**D**).

An example of the use of gas chromatography coupled to an MS detector for the determination of methanol generated in a carbon dioxide photocatalytic reduction using ^13^CO_2_ as a substrate is shown in Figure 10. The ^13^C-labeled and the non-labeled methanol have the same GC retention time, but the mass spectra for ^12^CH_3_OH and ^13^CH_3_OH, shown in Figure 10A,B, respectively, are different, allowing for a clear distinction between the two compounds and the identification of the ^13^C labeled samples. Figure 10C is the extracted ion chromatogram for methanol in photocatalytic experiments using non-labeled CO_2_, while Figure 10D is the extracted ion chromatogram in a test involving labeled ^13^CO_2_. In the chromatogram relevant to the use of ^13^CO_2_, peaks at m/z = 33 (^13^CH_3_OH), 31 (possible ^13^CH_2_O), and m/z = 29 (^13^CO) clearly show the origin of the carbon atom, thus evidencing that methanol is generated from ^13^CO_2_ and not from other C sources (lack of peaks at m/z 32, 31, 30, 28). NMR spectroscopy is very useful for the analysis of solutions of products, as labeled methanol has a signal well distinguished from that of ^12^C methanol.

Interestingly, it is questionable whether ^12^CO_2_ and ^13^CO_2_ have different kinetics in photocatalytic reactions. Barecka and co-authors reported that the electro-reduction of CO_2_ strongly favors the conversion of the ^12^C isotope of carbon in comparison with the ^13^C isotope [87]. Such behavior can be explained based on the general principle of lower activation energy for lighter iso-topologues in irreversible reactions. The ^13^C isotope is associated with lower zero-point energy and, in theory, higher energy is needed to break bonds in ^13^CO_2_ [87,88,89]. However, more confirmed evidence of the validity of such a principle regarding photocatalytic reactions is necessary.

### 3.3. Blank Test Importance

**Figure 11:**
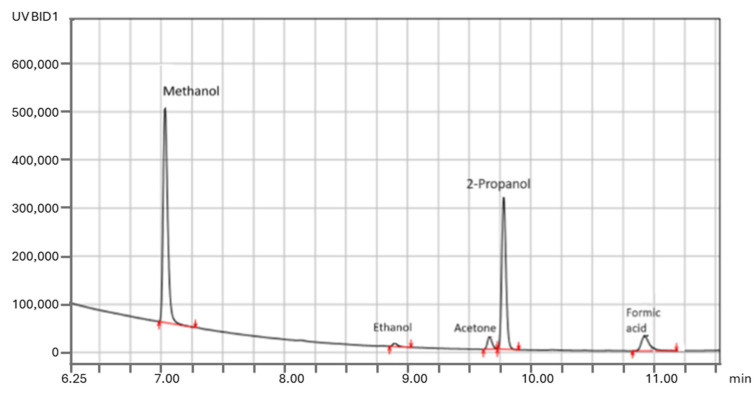
GC-BID analysis of the gas phase in equilibrium with liquid in a blank experiment performed by irradiation for 1 h with a Xe lamp of solid CuZnOx in an aqueous suspension under N_2_.

Despite the great advantages of experiments with labeled ^13^CO_2_, it should be highlighted that blank tests are the easiest and the most important tool for verifying experimental results. Blank experiments in the dark and/or in the absence of CO_2_ and/or catalyst are the most cost-effective strategy to understand the source of all observed products; therefore, they should be mandatory in photocatalytic CO_2_ conversion research [17]. The possible contribution from photocatalyst contaminations, as well as contaminations from photoreactors, glassware, reagents, and solvents, can be ruled out when an experiment is performed under an inert atmosphere (Ar or N_2_) instead of CO_2_. GC analysis of gas samples from blank tests during our control experiments indicated the presence of C_1_-C_5_ compounds such as methanol, acetone, isopropanol, and others (Figure 11), which originate from exogenous C instead of CO_2_. Performing such tests in the absence of CO_2_ or light allowed us to detect, identify, and quantify potential background contamination early and to avoid it, working in safe experimental conditions that allow for quantifying the reduction of CO_2_.

**Figure 12:**
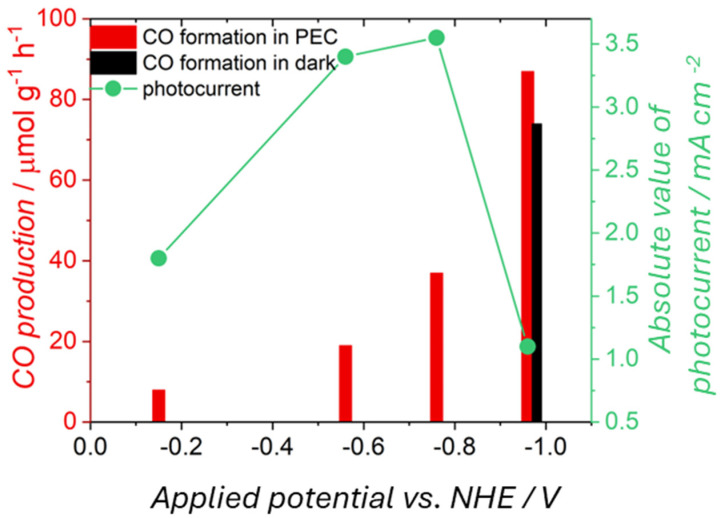
Photoelectrochemical reduction of carbon dioxide to carbon monoxide performed under various bias potentials using CuO@In_2_O_3_ as a photo(electro)catalyst. The left Y-axis refers to the efficiency of carbon monoxide formation, represented as bars. The right Y-axis corresponds to the absolute value of photocurrent density, shown as the green line. Tests were performed under light (red bars) or in the dark (black bars).

Noticeably, in dark experiments in the absence of CO_2_, any observed production of species like CO, methanol, methane, ethanol, and others can be attributed to a non-photocatalytic conversion of contaminants, undermining the claim that the observed products are due to photocatalytic CO_2_ reduction. Such experiments are also crucial for distinguishing between photocatalytic, photo-electrocatalytic, and electrocatalytic reduction of CO_2_. As an example, we discuss the copper–indium oxide electrodes case we recently reported [90]. The electrodes were tested in PEC conditions under different potential biases (Figure 12). With decreasing potential from −0.15 to −0.95 V vs. NHE, the amount of formed carbon monoxide from CO_2_ reduction increased significantly (from 19 to 86 mmol g^−1^ h^−1^). In comparison, the absolute value of photocurrent density (the differences between current upon light and current upon light pulse) did not follow the same trend. At the more negative value of potential, the formation of CO was the highest, while the photocurrent density was the lowest. A sudden drop in the density of generated photocurrents may be associated with the reduction of oxide materials to their metallic forms—Cu and In—which do not exhibit photoactivity. A test conducted in the dark (black bar in Figure 12) showed a comparable amount of produced CO as under illumination, indicating that the carbon monoxide formation occurred mainly via an electrocatalytic (more than photo-electro) pathway.

Finally, control experiments are necessary for validating experimental setups and analytical methodologies. They help to identify and rectify any systematic error or artifact that might affect the results.

For instance, the presence of leaks or the contamination of tools used in analyses of products can be detected and corrected through careful control experiments. This ensures that the reported experimental data are reliable and reproducible.

### 3.4. Good Practice in Analytical Procedures

Gas chromatography is the most widely used analytical tool to track CO_2_ reduction products. For accurate quantification and peak identification, it is crucial to ensure a clean background in a GC blank run, free from any interfering peaks or baseline changes that might affect measurements. This involves the following steps:

- Ensuring clean carrier/makeup gas and delivery tubing.
- Using clean solvents.
- Properly washing the syringe with proper solvents to prevent cross-contamination between samples.
- Checking the inlet liner/septum, column, and detector plumbing and base weldment for cleanliness.

When troubleshooting chromatographic issues occur, it is essential to conduct tests that may confine the problem to the detector, column, or sample introduction parts (inlet, liners, gas supply, and injection tool). In our laboratory, we use a high-sensitivity detector such as a BID (Barrier Discharge Ionization Detector) in CO2RP identification. This requires that all technical gases (carrier, makeup, and detector) meet specifications of 99.9999% purity. This standard allows for a maximum of 1 part per million (ppm) impurity. It is recommended to use gas traps for carrier and makeup gas supplies as follows: an all-metal conditioned moisture trap positioned closest to the tank, an all-glass O_2_ indicating trap positioned closest to the GC, and, optionally, a hydrocarbon trap installed in between.

## 4. Materials and Methods

### 4.1. Synthesis of Materials

#### 4.1.1. Synthesis of Rutin@TiO_2_

Titanium dioxide modified with rutin was prepared according to the method previously reported [39].

#### 4.1.2. Synthesis of SrTiO_3_

Deionized water and ethene glycol were mixed in a 1:1 volume ratio. Then, 6.8 mL of tetra-butyl titanate was dissolved in the above-mentioned solution (40 mL) and stirred for 30 min. Then, 20 mmol of strontium chloride was stirred in 20 mL potassium hydroxide solution (2 mol L^−1^) for 30 min. The solution of Sr was added dropwise to the solution of Ti under stirring. The resulting solution was transferred into a 200 mL stainless steel autoclave and heated at 220 °C for 12 h. The product was collected by centrifugation and washed with water until it reached a neutral pH and 3 times with ethanol. The washed products were dried under a vacuum over silica gel.

#### 4.1.3. Synthesis of ZnS Nanoparticles

First, 0.1 mol of zinc acetate was dissolved in 70 mL aqueous solution containing 7 mL of Triton x-100. The resulting mixture was heated under continuous stirring for 30 min at 60 °C. Subsequently, 30 mL of a solution containing 0.1 mol of Na_2_S was added, yielding a white suspension that was further stirred for 30 min at 60 °C to ensure homogeneous mixing. The resulting slurry was transferred into a flask equipped with a reflux condenser and heated at 120 °C for 10 h. The obtained precipitate was collected by centrifugation, washed with water, and dried at 60 °C in an oven.

#### 4.1.4. Synthesis of the CuZnOx Photocatalyst

A CuZnOx photocatalyst was obtained via the hydrothermal route using Cu(NO_3_)_2_ and Zn(NO_3_)_2_ as precursors, as previously described, and carefully washed and dried [91].

#### 4.1.5. Preparation of g-C_3_N_4_ Thin Film on a Glass Plate

g-C_3_N_4_ was prepared according to the literature protocol [92]. A thin film on a glass plate (2 × 5 cm) was prepared using a Fengda FE-183K airbrush with a 0.2 mm diameter nozzle. Then, 20 mg of g-C_3_N_4_ material was suspended in 4 mL of water, into which 0.4 mL of Nafion–isopropanol solution was added followed by sonication under stirring. The suspension was deposited on a glass plate by airbrush spray, with the glass plate placed on a heating plate kept at 80 °C for the duration of the deposition. Each glass plate piece was sprayed with 1 mL of suspension.

### 4.2. Photocatalytic Test

#### 4.2.1. Degradation of Triton X-100

First, 20 mg of photocatalyst (ZnS) was dispersed in 20 mL aqueous 0.5 mM solution of Triton X-100 by ultrasound agitation for a few minutes. Tests were performed under LED irradiation (λ = 420) in a vessel closed by a rubber septum. Gas samples were studied with a Nexis GC-2030, Shimadzu equipped with a Thermal Conductivity Detector (TCD) and a Barrier Discharge Ionization Detector (BID). Samples of the solution were collected periodically, filtered using syringe filters (0.22 μm), and analyzed using a Shimadzu UV-2600 UV-Vis spectrometer.

#### 4.2.2. Blank Test on Photocatalysts in the Absence of CO_2_

First, 10 mg of ZnS or CuZnO_x_ was suspended in 10 mL of water using ultrasound agitation for five minutes under N_2_. The suspension was then transferred under N_2_ into a glass vial equipped with a septum using a vacuum line. The vial was sealed under a N_2_ atmosphere and irradiated with a Xenon lamp for 1 h. After this period, the closed vial was heated at 90 °C for 10 min in an oven, and the vapor in equilibrium with liquid was withdrawn with a GC syringe and analyzed using gas chromatography (Nexis GC-2023 Shimadzu, equipped with TCD and BID detectors). Figure 11 shows that a number of organics were detected that are formed by the photodecomposition of residual organics present on the photocatalytic material.

#### 4.2.3. Photocatalytic Degradation of an Ionic Liquid

First, 10 mL of a 0.01 M aqueous solution of 1-butyl-3-methylimidazolium chloride (Sigma Aldrich, St. Louis, MO, USA) was irradiated using a Xe lamp for 30 min in the presence of CuZnO_x_ as photocatalyst (1 mg/mL). After this period, the vial was heated at 90 °C for 10 min and the vapors were analyzed using gas chromatography with a Nexis GC-2023 Shimadzu, equipped with TCD and BID detectors. The mass spectrum of the gas phase was obtained using a GC-MSQP-2010 SE-Shimadzu instrument.

#### 4.2.4. Photoelectrochemical Tests

A three-electrode quartz cell was used in photoelectrochemical measurements for applying a potential profile at the photocathode equivalent to linear voltammetry or constant potential. Photoelectrochemical measurements were controlled by a BioLogic SP-150 potentiostat. Copper indium cathodes [90] were the working electrode, and a silver chloride electrode (Ag-AgCl) and a platinum spiral were used as the reference and counter electrodes, respectively. The scan rate was 10 mV/s. An LED (λ = 400 nm) was used as a light source. The electrolyte (0.5 M KHCO_3_) was bubbled with nitrogen before each measurement. Electrochemical potentials were converted to the normal hydrogen electrode (NHE) scale. Tests were performed as a chronoamperometric run.

The products from the headspace above the reaction solution were analyzed by gas chromatography using a Nexis GC-2030, Shimadzu instrument equipped with TCD and BID.

### 4.3. Materials Characterization, Irradiation Experiments, and GC Analyses

FTIR spectra of frustules were measured using an IR-Spirit (Shimadzu) spectrophotometer with ATR attachment.

Energy-dispersive X-ray spectroscopy (EDS) analysis was performed using Scanning Electron Microscopy (SEM) using a Quanta FEG 250 microscope under low vacuum conditions at a pressure of 70 Pa with a beam accelerating voltage of 5 kV.

EDS analyses were carried out under low vacuum conditions at a pressure of 20 Pa with a beam accelerating voltage of 10 kV or 30 kV using the EDAX Octane SDD detector.

GC analyses were carried out with a Shimadzu GC 2030 equipped with a BID with a detection limit of 1 ppm.

NMR spectra were carried out with a Bruker 600 MHz. Mass spectra were recorded with a Shimadzu GCMS-QP2010.

Irradiation was carried out using a 300 W Xe lamp.

## 5. Conclusions

This article addresses the critical issue of false positive results in the photochemical and photoelectrochemical reduction of CO_2_. It identifies several potential sources of error, including the degradation of photocatalysts, the presence of contaminants from synthesis procedures, interference from laboratory materials, and environmental exposure.

Table 3 summarizes data relevant to the serendipitous exogenous sources of carbon and gives an idea of the incidence of false positives on the evaluation of photocatalysts and photoelectrocatalysts, comparing the target product concentration in contaminated and clean experiments. Very often, they are commensurable.

To mitigate such serious issues, the authors recommend stringent control measures such as thorough checking of the synthetic procedures of photocatalysts, eliminating any residual C-source, cleaning of photomaterials and devices from any possible contaminants, using isotopically labeled ^13^CO_2_, and conducting blank tests under inert gas (N_2_, Ar) in the dark and under light to verify that any possible exogenous C sources are absent. By implementing these practices, the accuracy and reliability of CO2RR experiments will be demonstrated, ultimately advancing the field of photocatalysis and enhancing the development of effective CO_2_ reduction technologies.

## Figures and Tables

**Table 1:** Keto–enol structure of acetylacetone and the products of photochemical decomposition.

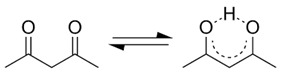			
Products	Percentage (%)	Products	Percentage (%)
Acetic acid	35	Acetaldehyde	2
Pyruvic acid	4	Carbon monoxide	20
Lactic acid	4	Formic acid	4
Formaldehyde	9	Others	22

**Table 2:** The concentration of isopropanol during a blank photocatalytic test using thin films prepared with a Nafion solution.

Conditions of Drying	Residual Concentration of Isopropanol on Materials
Room temperature for 12 h	895 ppm
80 °C for 2 h	574 ppm
150 °C for 2 h	18 ppm

**Table 3:** Incidence of false positives in the evaluation of photoactive materials.

Catalyst/Material/Source of Error	Origin of C Products	Detected Amount (False positive + CO2RP)	Real CO2RP Amount Clean Conditions
Rutin@TiO_2_	Rutin—organic sensitizer of photocatalyst	CO (88 mmol g^−1^) in a 3 h test	CO (9 mmol g^−1^) in a 3 h test
Polyetheneimine PEI	PEI used in the preparation of electrodes/materials	C1 and Cn carboxylic acids, aldehydes and ketones, amines, and imines	Absence of Cn products and N-derivatives
ZnS + Triton	Triton—surfactant used for the synthesis of photocatalyst	CO (693 mmol g^−1^ h^−1^), CH_4_ (95 mmol g^−1^ h^−1^), CH_3_OH (7 mmol g^−1^ h^−1^), C_2_H_5_OH (203 mmol g^−1^ h^−1^)	Not detected if no sacrificial electron donor
1-butyl-3-methylimidazolium/CuO/ZnO	1-butyl-3-methylimidazolium chloride—ionic liquid	CO (20 mmol g^−1^), CH_3_OH (46 mmol g^−1^), C_2_H_5_OH (11 mmol g^−1^), chlorobutane (134 mmol g^−1^)	CO (1.9 mmol g^−1^)
SrTiO_3_	Ethanol—solvent	C_2_H_5_OH (200 mmol g^−1^ h^−1^)	C_2_H_5_OH (0.6 mmol g^−1^ h^−1^)
g-C_3_N_4_	Nafion solution in isopropanol	Isopropanol from 14 to 0.3 mmol g^−1^ (depending on drying temperature)	Not detected
ZnS	Glycerol—e-donor	Formic acid (only qualitative analysis)	Formic acid (only minor)
Frustule	Organic contaminations	-	-
CuO@In_2_O_3_	Organic contaminations	CO formed in PEC (light + bias) 86 mmol g^−1^ h^−1^ CO formed in EC (bias) 73 mmol g^−1^ h^−1^	
Operator	Breath	Acetone, ethanol, methanol: concentrations up to 30 ppm were measured depending on the exposure time and health conditions of the operator. This amount is often well above (two or three times) the level of CO2RR acetone under controlled conditions.	

## Data Availability

All data are available from the open literature or reported in this paper.

## References

[1] Aresta M., Dibenedetto A., Aresta M., Dibenedetto A. (2021). Circular Economy and Carbon Dioxide Conversion. The Carbon Dioxide Revolution: Challenges and Perspectives for a Global Society.

[2] Summary of Global Climate Action at COP 28. https://unfccc.int/sites/default/files/resource/Summary_GCA_COP28.pdf.

[3] Sabri M.A., Al Jitan S., Bahamon D., Vega L.F., Palmisano G. (2021). Current and future perspectives on catalytic-based integrated carbon capture and utilization. Sci. Total Environ..

[4] Aresta M., Dibenedetto A. (2024). Merging the Green-H_2_ production with Carbon Recycling for stepping towards the Carbon Cyclic Economy. J. CO_2_ Util..

[5] Baran T., Wojtyła S., Dibenedetto A., Aresta M., Macyk W. (2015). Zinc sulfide functionalized with ruthenium nanoparticles for photocatalytic reduction of CO_2_. Appl. Catal. B Environ..

[6] Baran T., Visibile A., Busch M., He X., Wojtyla S., Rondinini S., Minguzzi A., Vertova A. (2021). Copper Oxide-Based Photocatalysts and Photocathodes: Fundamentals and Recent Advances. Molecules.

[7] Alkhatib I.I., Garlisi C., Pagliaro M., Al-Ali K., Palmisano G. (2020). Metal-organic frameworks for photocatalytic CO_2_ reduction under visible radiation: A review of strategies and applications. Catal. Today.

[8] Habisreutinger S.N., Schmidt-Mende L., Stolarczyk J.K. (2013). Photocatalytic Reduction of CO_2_ on TiO_2_ and Other Semiconductors. Angew. Chem. Int. Ed..

[9] Low J., Cheng B., Yu J., Jaroniec M. (2016). Carbon-based two-dimensional layered materials for photocatalytic CO_2_ reduction to solar fuels. Energy Storage Mater..

[10] Neaţu Ş., Maciá-Agulló J.A., Garcia H. (2014). Solar light photocatalytic CO_2_ reduction: General considerations and selected bench-mark photocatalysts. Int. J. Mol. Sci..

[11] Marszewski M., Cao S., Yu J., Jaroniec M. (2015). Semiconductor-based photocatalytic CO_2_ conversion. Mater. Horiz..

[12] Kumar A., Singh P., Khan A.A.P., Le Q.V., Nguyen V.-H., Thakur S., Raizada P. (2022). CO_2_ photoreduction into solar fuels via vacancy engineered bismuth-based photocatalysts: Selectivity and mechanistic insights. Chem. Eng. J..

[13] Sohail M., Anwar U., Taha T.A., Qazi H.I.A., Al-Sehemi A.G., Ullah S., Algarni H., Ahmed I.M., Amin M.A., Palamanit A. (2022). Nanostructured materials based on g-C_3_N_4_ for enhanced photocatalytic activity and potentials application: A review. Arab. J. Chem..

[14] Luo T., Gilmanova L., Kaskel S. (2023). Advances of MOFs and COFs for photocatalytic CO_2_ reduction, H_2_ evolution and organic redox transformations. Coord. Chem. Rev..

[15] Sadanandan A.M., Yang J.-H., Devtade V., Singh G., Dharmarajan N.P., Fawaz M., Lee J.M., Tavakkoli E., Jeon C.-H., Kumar P. (2024). Carbon nitride based nanoarchitectonics for nature-inspired photocatalytic CO_2_ reduction. Prog. Mater. Sci..

[16] Strunk J. (2023). Separating fiction from fact for photocatalytic CO_2_ reduction. Nat. Chem..

[17] Zhang K., Gao Q., Xu C., Zhao D., Zhu Q., Zhu Z., Wang J., Liu C., Yu H., Sun C. (2022). Current dilemma in photocatalytic CO_2_ reduction: Real solar fuel production or false positive outcomings?. Carbon Neutrality.

[18] Yang C.-C., Yu Y.-H., van der Linden B., Wu J.C.S., Mul G. (2010). Artificial Photosynthesis over Crystalline TiO_2_-Based Catalysts: Fact or Fiction?. J. Am. Chem. Soc..

[19] Grigioni I., Dozzi M.V., Bernareggi M., Chiarello G.L., Selli E. (2017). Photocatalytic CO_2_ reduction vs. H_2_ production: The effects of surface carbon-containing impurities on the performance of TiO_2_-based photocatalysts. Catal. Today.

[20] You J., Xiao M., Liu S., Lu H., Chen P., Jiang Z., Shangguan W., Wang Z., Wang L. (2023). How carbon contamination on the photocatalysts interferes with the performance analysis of CO_2_ reduction. J. Mater. Chem. A.

[21] Zhang Y., Yao D., Xia B., Jaroniec M., Ran J., Qiao S.-Z. (2022). Photocatalytic CO_2_ Reduction: Identification and Elimination of False-Positive Results. ACS Energy Lett..

[22] Wang S., Jiang B., Henzie J., Xu F., Liu C., Meng X., Zou S., Song H., Pan Y., Li H. (2023). Designing reliable and accurate isotope-tracer experiments for CO_2_ photoreduction. Nat. Commun..

[23] Ali S., Flores M.C., Razzaq A., Sorcar S., Hiragond C.B., Kim H.R., Park Y.H., Hwang Y., Kim H.S., Kim H. (2019). Gas Phase Photocatalytic CO_2_ Reduction, “A Brief Overview for Benchmarking”. Catalysts.

[24] Nosaka Y., Nosaka A.Y. (2017). Generation and Detection of Reactive Oxygen Species in Photocatalysis. Chem. Rev..

[25] Ola O., Maroto-Valer M.M. (2015). Review of material design and reactor engineering on TiO_2_ photocatalysis for CO_2_ reduction. J. Photochem. Photobiol. C Photochem. Rev..

[26] Shoji S., Yamaguchi A., Sakai E., Miyauchi M. (2017). Strontium Titanate Based Artificial Leaf Loaded with Reduction and Oxidation Cocatalysts for Selective CO_2_ Reduction Using Water as an Electron Donor. ACS Appl. Mater. Interfaces.

[27] Navalón S., Dhakshinamoorthy A., Álvaro M., Garcia H. (2013). Photocatalytic CO_2_ reduction using non-itanium metal oxides and sulfides. ChemSusChem.

[28] Baran T., Wojtyła S., Dibenedetto A., Aresta M., Macyk W. (2016). Photocatalytic Carbon Dioxide Reduction at p-Type Copper(I) Iodide. ChemSusChem.

[29] Khaidar D.M., Isahak W.N.R.W., Ramli Z.A.C., Ahmad K.N. (2024). Transition metal dichalcogenides-based catalysts for CO_2_ conversion: An updated review. Int. J. Hydrogen Energy.

[30] Alshamkhani M.T., Teong L.K., Putri L.K., Mohamed A.R., Lahijani P., Mohammadi M. (2021). Effect of graphite exfoliation routes on the properties of exfoliated graphene and its photocatalytic applications. J. Environ. Chem. Eng..

[31] Domingo-Tafalla B., Martínez-Ferrero E., Franco F., Palomares-Gil E. (2022). Applications of Carbon Dots for the Photocatalytic and Electrocatalytic Reduction of CO_2_. Molecules.

[32] Sinopoli A., La Porte N.T., Wasielewski M.R., Sohail M. (2018). Photosensitisers for CO_2_ photoreduction: From metal complexes to rylenes, an overview. ACS Symp. Ser..

[33] Bizzarri C. (2022). Homogeneous Systems Containing Earth-Abundant Metal Complexes for Photoactivated CO_2_ Reduction: Recent Advances. Eur. J. Org. Chem..

[34] Cheong H.-Y., Kim S.-Y., Cho Y.-J., Cho D.W., Kim C.H., Son H.-J., Pac C., Kang S.O. (2017). Photosensitization Behavior of Ir(III) Complexes in Selective Reduction of CO_2_ by Re(I)-Complex-Anchored TiO_2_ Hybrid Catalyst. Inorg. Chem..

[35] Kumagai H., Tamaki Y., Ishitani O. (2022). Photocatalytic Systems for CO_2_ Reduction: Metal-Complex Photocatalysts and Their Hybrids with Photofunctional Solid Materials. Acc. Chem. Res..

[36] Ozcan O., Yukruk F., Akkaya E.U., Uner D. (2007). Dye sensitized CO_2_ reduction over pure and platinized TiO_2_. Top. Catal..

[37] Shizuno M., Kato K., Nishioka S., Kanazawa T., Saito D., Nozawa S., Yamakata A., Ishitani O., Maeda K. (2022). Effects of a Nanoparticulate TiO_2_ Modifier on the Visible-Light CO_2_ Reduction Performance of a Metal-Complex/Semiconductor Hybrid Photocatalyst. ACS Appl. Energy Mater..

[38] Buchalska M., Kuncewicz J., Świętek E., Łabuz P., Baran T., Stochel G., Macyk W. (2013). Photoinduced hole injection in semiconductor-coordination compound systems. Coord. Chem. Rev..

[39] Labuz P., Sadowski R., Stochel G., Macyk W. (2013). Visible light photoactive titanium dioxide aqueous colloids and coatings. Chem. Eng. J..

[40] Aresta M., Dibenedetto A., Baran T., Angelini A., Labuz P., Macyk W. (2014). An integrated photocatalytic/enzymatic system for the reduction of CO_2_ to methanol in bioglycerol-water. Beilstein J. Org. Chem..

[41] Ramanathan S., Thamilselvan A., Radhika N., Padmanabhan D., Durairaj A., Obadiah A., Lydia I.S., Vasanthkumar S. (2021). Development of rutin-rGO/TiO_2_ nanocomposite for electrochemical detection and photocatalytic removal of 2,4-DCP. J. Iran. Chem. Soc..

[42] Marchal C., Behr M., Vigneron F., Caps V., Keller V. (2016). Au/TiO_2_ photocatalysts prepared by solid grinding for artificial solar-light water splitting. New J. Chem..

[43] Tsunoji N., Ide Y., Yagenji Y., Sadakane M., Sano T. (2014). Design of Layered Silicate by Grafting with Metal Acetylacetonate for High Activity and Chemoselectivity in Photooxidation of Cyclohexane. ACS Appl. Mater. Interfaces.

[44] Habibi M.H., Askari E. (2013). Spectrophotometric studies of photo-induced degradation of Tertrodirect Light Blue (TLB) using a nanostructure zinc zirconate composite. J. Ind. Eng. Chem..

[45] Wu B., Zhang G., Zhang S. (2016). Fate and implication of acetylacetone in photochemical processes for water treatment. Water Res..

[46] Thangamuthu M., Ruan Q., Ohemeng P.O., Luo B., Jing D., Godin R., Tang J. (2022). Polymer Photoelectrodes for Solar Fuel Production: Progress and Challenges. Chem. Rev..

[47] Hidalgo D., Bocchini S., Fontana M., Saracco G., Hernández S. (2015). Green and low-cost synthesis of PANI–TiO_2_ nanocomposite mesoporous films for photoelectrochemical water splitting. RSC Adv..

[48] Mohammed A.M., Mohtar S.S., Aziz F., Aziz M., Ul-Hamid A. (2021). Cu_2_O/ZnO-PANI ternary nanocomposite as an efficient photocatalyst for the photodegradation of Congo Red dye. J. Environ. Chem. Eng..

[49] Wang X., Shen Y., Xie A., Qiu L., Li S., Wang Y. (2011). Novel structure CuI/PANI nanocomposites with bifunctions: Superhydrophobicity and photocatalytic activity. J. Mater. Chem..

[50] Wang W., Xu J., Zhang L., Sun S. (2014). Bi_2_WO_6_/PANI: An efficient visible-light-induced photocatalytic composite. Catal. Today.

[51] Wang Y., Li S., Shi H., Yu K. (2012). Facile synthesis of p-type Cu_2_O/n-type ZnO nano-heterojunctions with novel photoluminescence properties, enhanced field emission and photocatalytic activities. Nanoscale.

[52] Bai X., Luan J., Song T., Sun H., Yan B., Dai Y., Yu J. (2024). Polyvinyl alcohol/polyethyleneimine grafted carbon oxynitride composite nanofiber membranes with the synergistical enhanced photocatalytic degradation and bactericidal performance. J. Appl. Polym. Sci..

[53] Ben-Shahar Y., Scotognella F., Waiskopf N., Kriegel I., Conte S.D., Cerullo G., Banin U. (2015). Effect of Surface Coating on the Photocatalytic Function of Hybrid CdS–Au Nanorods. Small.

[54] Coralli I., Fabbri D., Facchin A., Torri C., Stevens L.A., Snape C.E. (2023). Analytical pyrolysis of polyethyleneimines. J. Anal. Appl. Pyrolysis.

[55] Liang Q., Liu X., Zeng G., Liu Z., Tang L., Shao B., Zeng Z., Zhang W., Liu Y., Cheng M. (2019). Surfactant-assisted synthesis of photocatalysts: Mechanism, synthesis, recent advances and environmental application. Chem. Eng. J..

[56] Han D., Yang H., Zhu C., Wang F. (2008). Controlled synthesis of CuO nanoparticles using TritonX-100-based water-in-oil reverse micelles. Powder Technol..

[57] Lu H., Ju H., Yang Q., Li Z., Ren H., Xin X., Xu G. (2013). Synthesis of Ag@SiO_2_ hybrid nanoparticles templated by a Triton X-100)/1-hexanol/cyclohexane/H_2_O water-in-oil microemulsion. CrystEngComm.

[58] Matejka P., Vlckova B., Vohlidal J., Pancoska P., Baumruk V. (1992). The role of triton X-100 as an adsorbate and a molecular spacer on the surface of silver colloid: A surface-enhanced Raman scattering study. J. Phys. Chem..

[59] Rahman F., Hossain J., Kuddus A., Moon M.A., Ismail A.B. (2020). Effect of Triton X-100 surfactant on thiol-amine cosolvents assisted facile synthesized CdS thin films on glass substrate by spin coating method. SN Appl. Sci..

[60] Salili S.M., Worden M., Nemati A., Miller D.W., Hegmann T. (2017). Synthesis of Distinct Iron Oxide Nanomaterial Shapes Using Lyotropic Liquid Crystal Solvents. Nanomaterials.

[61] Saien J., Ojaghloo Z., Soleymani A.R., Rasoulifard M.H. (2011). Homogeneous and heterogeneous AOPs for rapid degradation of Triton X-100 in aqueous media via UV light, nano titania hydrogen peroxide and potassium persulfate. Chem. Eng. J..

[62] Corchero R., Rodil R., Soto A., Rodil E. (2021). Nanomaterial Synthesis in Ionic Liquids and Their Use on the Photocatalytic Degradation of Emerging Pollutants. Nanomaterials.

[63] Hammond O.S., Mudring A.-V. (2022). Ionic liquids and deep eutectics as a transformative platform for the synthesis of nanomaterials. Chem. Commun..

[64] Ambika S., Sundrarajan M. (2016). [EMIM] BF4 ionic liquid-mediated synthesis of TiO_2_ nanoparticles using *Vitex negundo Linn* extract and its antibacterial activity. J. Mol. Liq..

[65] Buettner C.S., Cognigni A., Schröder C., Bica-Schröder K. (2022). Surface-active ionic liquids: A review. J. Mol. Liq..

[66] Klähn M., Stüber C., Seduraman A., Wu P. (2010). What Determines the Miscibility of Ionic Liquids with Water? Identification of the Underlying Factors to Enable a Straightforward Prediction. J. Phys. Chem. B.

[67] Zhu L., Chen Y., Sun Y., Cui Y., Liang M., Zhao J., Li N. (2010). Phase-manipulable synthesis of Cu-based nanomaterials using ionic liquid 1-butyl-3-methyl-imidazole tetrafluoroborate. Cryst. Res. Technol..

[68] Baran T. (2023). Efficiency of volatile organic compound degradation in air using doped strontium titanate photocatalysts. Quenching experiments towards understanding of doping mechanisms. React. Kinet. Mech. Catal..

[69] Yadav M., Gyulavári T., Kiss J., Ábrahámné K.B., Efremova A., Szamosvölgyi Á., Pap Z., Sápi A., Kukovecz Á., Kónya Z. (2023). Noble metal nanoparticles and nanodiamond modified strontium titanate photocatalysts for room temperature CO production from direct hydrogenation of CO_2_. J. CO_2_ Util..

[70] Burek B.O., Timm J., Bahnemann D.W., Bloh J.Z. (2019). Kinetic effects and oxidation pathways of sacrificial electron donors on the example of the photocatalytic reduction of molecular oxygen to hydrogen peroxide over illuminated titanium dioxide. Catal. Today.

[71] Pellegrin Y., Odobel F. (2017). Sacrificial electron donor reagents for solar fuel production. Comptes Rendus Chim..

[72] Dibenedetto A., Stufano P., Macyk W., Baran T., Fragale C., Costa M., Aresta M. (2012). Hybrid technologies for an enhanced carbon recycling based on the enzymatic reduction of CO_2_ to methanol in water: Chemical and photochemical NADH regeneration. ChemSusChem.

[73] Johne P., Kisch H. (1997). Photoreduction of carbon dioxide catalysed by free and supported zinc and cadmium sulphide powders. J. Photochem. Photobiol. A Chem..

[74] Wishart D.S., Knox C., Guo A.C., Eisner R., Young N., Gautam B., Hau D.D., Psychogios N., Dong E., Bouatra S. (2009). HMDB: A knowledgebase for the human metabolome. Nucleic Acids Res..

[75] Moret S., Dyson P.J., Laurenczy G. (2013). Direct, in situ determination of pH and solute concentrations in formic acid dehydrogenation and CO_2_ hydrogenation in pressurised aqueous solutions using ^1^H and ^13^C NMR spectroscopy. Dalton Trans..

[76] Wojtyła S., Klama P., Baran T. (2017). Is 3D printing safe? Analysis of the thermal treatment of thermoplastics: ABS, PLA, PET, and nylon. J. Occup. Environ. Hyg..

[77] Wojtyła S., Klama P., Śpiewak K., Baran T. (2019). 3D printer as a potential source of indoor air pollution. Int. J. Environ. Sci. Technol..

[78] Jones N.R., de Jersey A.M., Lavers J.L., Rodemann T., Rivers-Auty J. (2024). Identifying laboratory sources of microplastic and nanoplastic contamination from the air, water, and consumables. J. Hazard. Mater..

[79] McDonald G.R., Hudson A.L., Dunn S.M.J., You H., Baker G.B., Whittal R.M., Martin J.W., Jha A., Edmondson D.E., Holt A. (2008). Bioactive Contaminants Leach from Disposable Laboratory Plasticware. Science.

[80] Mochalski P., King J., Unterkofler K., Amann A. (2013). Stability of selected volatile breath constituents in Tedlar, Kynar and Flexfilm sampling bags. Analyst.

[81] Idris S.A.A., Hanafiah M.M., Ismail M., Abdullah S., Khan M.F. (2020). Laboratory air quality and microbiological contamination in a university building. Arab. J. Geosci..

[82] Yau Y.H., Chew B.T., Saifullah A.Z.A. (2012). Studies on the indoor air quality of Pharmaceutical Laboratories in Malaysia. Int. J. Sustain. Built Environ..

[83] Seseña S., Rodríguez A.M., Palop M.L. (2022). Indoor air quality analysis in naturally ventilated university training laboratories: A health risk assessment. Air Qual. Atmos. Health.

[84] Sharma A., Kumar R., Varadwaj P. (2023). Smelling the Disease: Diagnostic Potential of Breath Analysis. Mol. Diagn. Ther..

[85] Wasse S., Baran T., Mesto E., Norici A., Dibenedetto A., Aresta M. Formation of faceted [111, 110] Cu_2_O induced by Frustules and their activity as photo(electro)catalysts for the coprocessing of CO_2_&H_2_O to energy products under Visible-light irradiation. Proceedings of the 20th International Conference on Carbon Dioxide Utilization (ICCDU).

[86] Donose B.C., Taran E., Vakarelski I.U., Shinto H., Higashitani K. (2006). Effects of cleaning procedures of silica wafers on their friction characteristics. J. Colloid Interface Sci..

[87] Barecka M.H., Kovalev M.K., Muhamad M.Z., Ren H., Ager J.W., Lapkin A.A. (2023). CO_2_ electroreduction favors carbon isotope 12C over 13C and facilitates isotope separation. iScience.

[88] Roth J.P., Klinman J.P., Lennarz W.J., Lane M.D. (2004). Kinetic Isotope Effects. Encyclopedia of Biological Chemistry.

[89] Bard A.J. (1974). Encyclopedia of Electrochemistry of the Elements.

[90] Wojtyła S., Baran T. (2020). Electrochemically prepared copper/indium oxides photocathode for efficient photoelectrochemical hydrogen production. Sol. Energy Mater. Sol. Cells.

[91] Wojtyła S., Baran T. (2019). Copper zinc oxide heterostructure nanoflowers for hydrogen evolution. Int. J. Hydrogen Energy.

[92] Wojtyła S., Szmit K., Baran T. (2018). Type II Heterostructures: The Way Towards Improved Photoelectrochemical Activity of Graphitic Carbon Nitride. J. Inorg. Organomet. Polym..

